# Plasma Levels of Monocyte Chemotactic Protein-1 Are Associated with Clinical Features and Angiogenesis in Patients with Multiple Myeloma

**DOI:** 10.1155/2016/7870590

**Published:** 2016-01-27

**Authors:** Toni Valković, Emina Babarović, Ksenija Lučin, Sanja Štifter, Merica Aralica, Irena Seili-Bekafigo, Antica Duletić-Načinović, Nives Jonjić

**Affiliations:** ^1^Department of Hematology, Rijeka University Hospital Centre, Krešimirova 42, 51000 Rijeka, Croatia; ^2^Department of Pathology, School of Medicine, University of Rijeka, Braće Branchetta 20, 51000 Rijeka, Croatia; ^3^Department of Laboratory Medicine, Rijeka University Hospital Centre, Krešimirova 42, 51000 Rijeka, Croatia; ^4^Department of Cytology, Rijeka University Hospital Centre, Krešimirova 42, 51000 Rijeka, Croatia

## Abstract

The aim of this pilot study was to determine the plasma levels of monocyte chemotactic protein-1 (MCP-1) and possible associations with angiogenesis and the main clinical features of untreated patients with multiple myeloma (MM). ELISA was used to determine plasma MCP-1 levels in 45 newly diagnosed MM patients and 24 healthy controls. The blood vessels were highlighted by immunohistochemical staining, and computer-assisted image analysis was used for more objective and accurate determination of two parameters of angiogenesis: microvessel density (MVD) and total vascular area (TVA). The plasma levels of MCP-1 were compared to these parameters and the presence of anemia, renal dysfunction, and bone lesions. A significant positive correlation was found between plasma MCP-1 concentrations and TVA (*p* = 0.02). The MCP-1 levels were significantly higher in MM patients with evident bone lesions (*p* = 0.01), renal dysfunction (*p* = 0.02), or anemia (*p* = 0.04). Therefore, our preliminary results found a positive association between plasma MCP-1 levels, angiogenesis (expressed as TVA), and clinical features in patients with MM. However, additional prospective studies with a respectable number of patients should be performed to authenticate these results and establish MCP-1 as a possible target of active treatment.

## 1. Introduction

Multiple myeloma (MM) represents a common hematological neoplasm characterized by monoclonal expansion of plasma cells within the bone marrow, production of monoclonal immunoglobulins, and tissue impairment. The unpredictable biological behavior of this neoplasm reflects complex interactions between plasma cells and other components of the bone marrow microenvironment. Despite great improvements in therapy and significant prolongation of life expectancy, MM remains an incurable disease [1].

The limited success achieved by targeting only myeloma cells in conventional and/or high-dose chemotherapy highlights the importance of understanding the role of the bone marrow microenvironment and its specific contribution to myelomagenesis. In MM, the microenvironment is composed of clonal plasma cells, extracellular matrix proteins, bone marrow stromal cells, inflammatory cells, and microvessels. Substantial evidence indicates that interactions between these components play a key role in the proliferation and survival of myeloma cells, angiogenic and osteoclastogenic processes, and the development of drug resistance, which all lead to disease progression [2]. The antimyeloma activity of proteasome inhibitors (bortezomib, carfilzomib) and immunomodulatory drugs (thalidomide, lenalidomide, and pomalidomide) is based on their capacity to disrupt these pathophysiological processes [3, 4].

Angiogenesis is fundamental to tumor growth and spread in many hematological disorders, particularly MM [5]. The angiogenic potential of MM is regulated by a plethora of proangiogenesis and antiangiogenesis cytokines produced by myeloma cells and other cell types in the tumor microenvironment [6].

Among the many biologically active factors produced by the MM microenvironment are chemokines and their receptors, which participate in cell homing, attraction of leukocytes, tumor growth, and bone destruction [7, 8]. One of the CC chemokines secreted by MM cells is monocyte chemotactic protein-1 (MCP-1), which acts as a potent chemoattractant for monocytes, basophils, eosinophils, endothelial cells, a subset of T lymphocytes, and myeloma cells through its CCR2 receptor [9, 10]. In addition, MCP-1 is the first CC chemokine reported to play a direct role in tumor angiogenesis [11]. However, no studies have yet explored associations between plasma MCP-1 levels, angiogenesis, and the main clinical features in newly diagnosed, untreated myeloma patients, such as anemia, renal dysfunction, and bone disease, which was the aim of the present pilot study.

## 2. Methods

### 2.1. Patients

We retrospectively analyzed 45 newly diagnosed, previously untreated myeloma patients (22 males, 23 females; median age 69 years; age range 44–86 years) and 24 age-matched healthy individuals as a control group (12 males, 12 females; median age 67 years; age range 35–83 years). Diagnoses were established at the Department of Hematology, Clinical Centre Rijeka, between 2010 and 2012 according to the International Myeloma Working Group Criteria [12]. The main characteristics of the patients are summarized in Table 1.

The clinical parameters at the time of diagnosis were anemia (hemoglobin 20 g/L below the lower limit of normal, defined as 138 g/L for men and 119 g/L for women), renal dysfunction (serum creatinine level above the upper limit of normal, defined as 117 *μ*mol/L for men and 96 *μ*mol/L for women), and bone disease (the presence of any lytic lesion or severe osteopenia with compressive fractures on standard bone radiographs). The study was approved by the local ethics committees.

### 2.2. Immunohistochemistry

Bone marrow biopsies (BMBs) from our myeloma patients were fixed in Schaffer fixative for 24 hours and decalcinated in osteodec (Bio-Optica, Milan, Italy) for 4-5 hours. Sections were stained with hematoxylin-eosin, Giemsa, periodic acid Schiff (PAS), Prussian blue, and Gomori's stain for reticulin fibers. Sections of paraffin embedded BMB samples were processed for immunohistochemical analysis in a Dako Autostainer Plus (DakoCytomation Colorado, Fort Collins, CO, USA) according to the manufacturer's protocol using the Envision procedure (DAKO EnVision FLEX, High pH KIT K801021, Glostrup, Denmark). Samples were routinely immunohistochemically stained with anti-CD138 (clone MI15, m7228, DAKO Glostrup, Denmark), Ig kappa (number 40191, DAKO, Glostrup, Denmark), and Ig lambda (number 40193, DAKO, Glostrup, Denmark) antibodies for detection of the monoclonal antibody anti-CD34 Class II (m7165 clone QBEnd10, DAKO, Glostrup, Denmark), which was used to highlight endothelial cells. Epitope retrieval was achieved by immersing slides in Tris-EDTA buffer (pH 9.0) and boiling for 15 minutes in a water bath at 97°C. The slides were then incubated with CD34 monoclonal antibody at 1 : 100 dilution for 30 minutes at room temperature. For negative controls, a limited number of cases were stained by substituting primary antibody with buffer solution (DAKO).

### 2.3. Evaluation of Immunostaining

**Figure 1:**
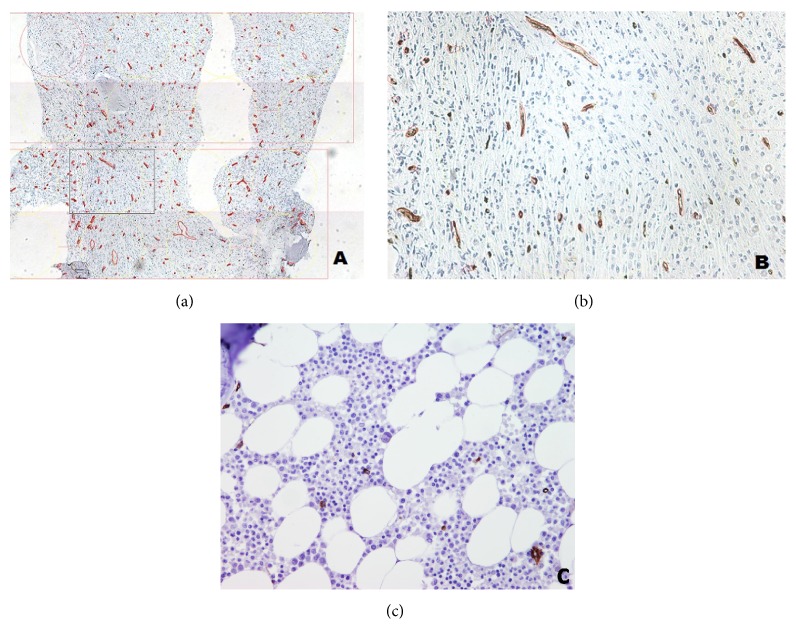
Computer-assisted image analysis (CIA) of the average number of microvessels per 1 mm^2^, referred to as the microvessel density (MVD) as shown on (a), and the percentage of microvessel area in the total section area, or the total vascular area (TVA) on (b). For comparison normal bone marrow (×100) stained with CD34 is attached on (c).

All slides stained with anti-CD34 were scanned and analyzed using the Alphelys Spot Browser 2 integrated system and software-controlled (Alphelys Spot Browser 2.4.4., France) stage positioning of a Nikon Eclipse 50i microscope mounted with a 1360 × 1024 resolution Microvision CFW-1310C digital camera as described previously [13]. Computer-assisted image analysis (CIA) was used for a more objective and accurate determination of angiogenic parameters. Briefly, during digital image analysis, the software detected objects of interest based on pixel color properties (wavelength, intensity, and saturation), grouping, and morphometry (size and shape). These measurements were used to calculate the average number of microvessels per 1 mm^2^, referred to as the microvessel density (MVD), and the percentage of microvessel area in the total section area or the total vascular area (TVA) (Figures 1(a) and 1(b)).

### 2.4. Measurement of MCP-1 in Plasma

The concentration of MCP-1 was measured in plasma samples by enzyme-linked immunoassay (ELISA; Quantikine R&D Systems, Minneapolis, MN, USA) according to the manufacturer's instructions. These assays employ the quantitative sandwich immunoassay technique. The optical density of each well was measured using the microplate reader set at 450 nm. The concentration of MCP-1 in each plasma sample was calculated from standard curves and reported in picograms per milliliter.

### 2.5. Statistical Analysis

Statistical analyses were performed using MedCalc for Windows, version 12.2.1.0 (MedCalc Software, Ostend, Belgium). The distribution of data was tested for normality using the Kolmogorov-Smirnov test. The Mann-Whitney *U* test was used to assess whether MCP-1 plasma concentrations differed significantly between categories: patients with bone lesions versus patients without bone lesions, patients with renal dysfunction versus patients without renal dysfunction, and patients with anemia versus patients without anemia. Correlations between MCP-1 and angiogenic parameters (MVD and TVA) were studied using the Pearson correlation. Statistical differences with *p* < 0.05 were considered significant.

## 3. Results

**Figure 2:**
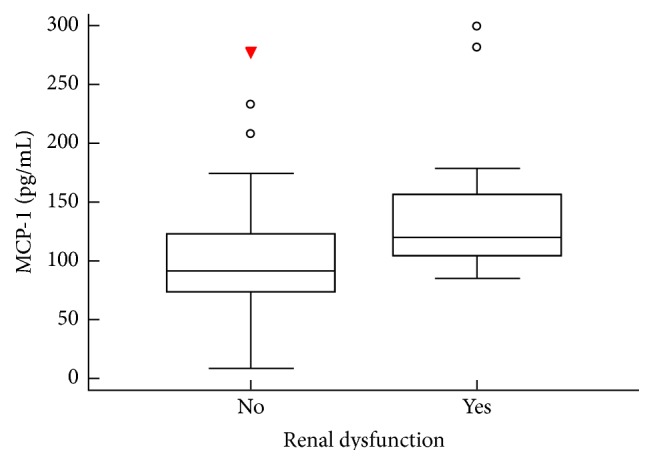
Comparison of plasma MCP-1 levels between patients who had normal creatinine values and those with renal dysfunction. The plasma concentration of MCP-1 was significantly higher in patients with renal dysfunction (*p* = 0.02, Mann-Whitney *U* test). The bars indicate the 75th and 25th percentiles, and the line in each box represents the median.

**Figure 3:**
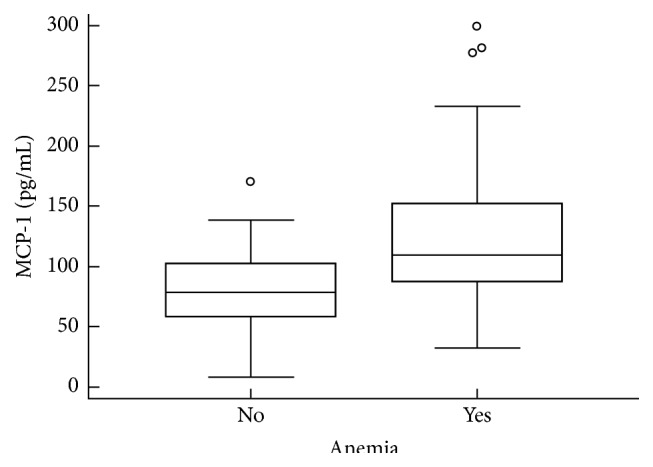
Comparison of plasma MCP-1 levels between patients without anemia and patients with anemia. The plasma concentration of MCP-1 was significantly higher in patients with anemia (*p* = 0.04, Mann-Whitney *U* test). The bars indicate the 75th and 25th percentiles, and the line in each box represents the median.

**Figure 4:**
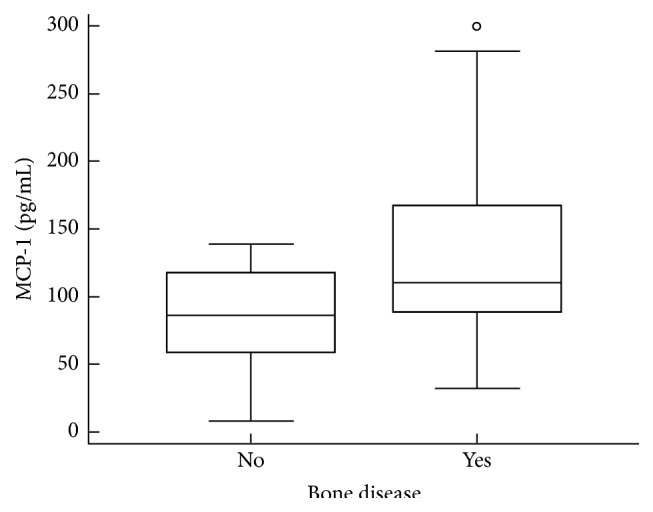
Comparison of plasma MCP-1 levels between patients with bone disease and those without bone lesions. The plasma concentration of MCP-1 was significantly higher in patients with bone disease (*p* = 0.01, Mann-Whitney *U* test). The bars indicate the 75th and 25th percentiles, and the line in each box represents the median.

MCP-1 was detected in plasma samples from all patients and healthy controls, and no significant differences were found between MM patients (median 105.6 pg/mL, range 8.3–299.5 pg/mL) and healthy controls (median 103.5 pg/mL, range 69.5–175.2 pg/mL; *p* = 0.83). Plasma MCP-1 levels were significantly higher in patients with renal dysfunction (median 120.3 pg/mL, range 84.7–299.5 pg/mL) in comparison with patients who had no renal impairment (median 91.5 pg/mL, range 8.3–277.4 pg/mL; *p* = 0.02; Figure 2). Likewise, plasma MCP-1 levels were higher in patients with anemia (median 109.5 pg/mL, range 32.1–299.5 pg/mL) in comparison with patients who had normal hemoglobin values (median 78.9 pg/mL, range 8.3–170.2 pg/mL; *p* = 0.04; Figure 3). Furthermore, patients with evident bone lesions had significantly higher concentrations of plasma MCP-1 compared to patients without the presence of any lytic lesion or severe osteopenia with compressive fractures on standard bone radiographs (median 110.3 pg/mL, range 32.1–299.5 pg/mL versus median 86.4 pg/mL, range 8.3–138.9 pg/mL; *p* = 0.01; Figure 4 and Table 2).

Angiogenic parameters for the patient cohort were as follows: median MVD was 179 (range 42–685) and median TVA was 2.09% (range 0.41%–17.3%). Comparison of the plasma MCP-1 levels and angiogenic parameters in patient BMBs yielded the following results: there was a significant positive correlation between plasma MCP-1 concentrations and TVA (*r* = 0.347, *p* = 0.02), but no significant correlation was found regarding MCP-1 and MVD (*r* = 0.207, *p* = 0.18; Table 3).

## 4. Discussion

Our current research is a continuation of the series of pilot studies attempting to identify potentially important cytokines influencing MM. Our previous preliminary results indicated a positive association between plasma levels of OPN, bone destruction, and tumor burden, suggesting that OPN can be a useful biomarker for monitoring bone disease and tumor mass [14].

Bone marrow angiogenesis increases with disease progression across the spectrum of plasma cell dyscrasias [15, 16]. Furthermore, parameters of angiogenesis have been established as adverse prognostic factors for MM survival [17–19], correlating with other prognosticators [15, 16, 20, 21]. Novel agents against MM, such as proteasome inhibitors or immunomodulatory drugs that significantly improve the response to therapy and survival, exhibit marked antiangiogenic effects. However, we do not yet know which of these numerous soluble factors play central roles in the regulation of angiogenesis. Salcedo et al. demonstrated that MCP-1 may directly induce blood vessel formation* in vivo*, which can be inhibited using neutralizing antibodies against MCP-1 [11]. Niu et al. showed that MCP-1 promotes angiogenesis* via* a transcription factor, MCP-1-induced protein [22]. To the best of our knowledge, the current pilot study is the first study concerning possible correlations between plasma levels of MCP, parameters of angiogenesis, and clinical manifestations in patients with MM. The presence of MCP-1 in plasma samples from all patients and healthy controls implicates this chemokine in physiological and pathological processes. Even though the patients had slightly higher concentrations of MCP-1 than controls, the difference was not significant. This finding can be attributed to the small number of samples. Until now, only a few studies of MM have explored both MVD and TVA. Rana et al. found a significant correlation between MVD and TVA, both of which correlated with other examined histological features associated with prognosis and residual disease in myeloma patients [23]. Bhatti et al. also demonstrated a good correlation between MVD and TVA; “complete responders” had significantly less angiogenesis than “nonresponders,” but only MVD was a good predictor of a complete response in patients with MM, particularly when the analysis was performed using a computerized image analyzer [24]. Tzenou et al. evaluated angiogenesis in trephine biopsy specimens from 36 patients with Waldenström's macroglobulinemia. Only TVA, not MVD, significantly correlated with time to first therapy and overall survival [25]. Our results show for the first time a positive correlation between plasma MCP-1 levels and angiogenesis in myeloma patients, as patients with higher plasma MCP-1 levels had significantly higher TVA in BMBs, whereas MVD failed to show a significant association with chemokine concentrations. The reason for this finding is questionable. Of course, it may be a consequence of the rather small number of patients; therefore, this result should be retested in a larger sample. However, it is doubtful whether these two angiogenic parameters actually provide the same biological information. The magnitude of MVD is determined mainly by the number of small blood vessels, many of which still do not have a formed vascular lumen. On the other hand, TVA is defined as the percentage of microvessel area in the total section area, meaning that the presence of larger vessels with formed vascular lumen increases the value of TVA more than the number of small blood vessels. Further investigations are needed to fully clarify this issue.

Our preliminary results demonstrated a significant association between plasma MCP-1 levels and the main clinical features of MM. Namely, patients with higher chemokine levels exhibited more severe bone disease, renal impairment, and anemia. The association between increased plasma level of MCP-1 and creatinine concentration should be taken with caution because it may be the result of decreased renal clearance of this chemokine in patients with renal failure.

We hypothesized that, in myeloma patients, proinflammatory cytokine tumor necrosis factor-alpha (TNF-*α*) upregulates the production of interleukin-6 (IL-6), which then mediates increased secretion of MCP-1. These three biologically active factors produced by myeloma cells and other cellular elements of the bone marrow microenvironment can influence the activity and survival of osteoclasts and osteoblasts and increase inflammatory processes in the bone marrow and kidneys, leading to anemia and renal disease. The role of TNF-*α* and IL-6 in the progression of myeloma cell growth, survival, angiogenesis, and osteoclastogenesis and the inhibition of osteoblast activity is well established, as well as their adverse prognostic significance in this hematological neoplasm [8, 26–30]. Lee et al. reported that the level of IL-6 in bone marrow aspirates from myeloma patients positively correlates with the level of TNF-*α*, and these cytokines correlated with poor prognostic factors and short overall survival [30]. Lee et al. also demonstrated that IL-6 secretion is regulated by TNF-*α* via the JAK/STAT pathway in U266 myeloma cells [30]. Arendt et al. found that IL-6 induces MCP-1 expression in myeloma cells, suggesting a new mechanism by which IL-6 may contribute to disease pathogenesis [31]. Johrer et al. reported that transendothelial migration of myeloma cells is increased by TNF-*α* via TNF receptor 2 and autocrine upregulation of MCP-1, demonstrating again the possible mutual relationship between these cytokines and chemokines [32]. Speaking of bone disease in MM, Liu et al. showed that MM cells increase the production of MCP-1 by bone marrow stromal cells, which then enhances osteoclast formation [33]. Although the current study has limitations, such as the small sample size, missing complete clinical data for one patient, and retrospective design, that limit any strong conclusions, our preliminary results indicate a positive association between plasma MCP-1 levels, angiogenic parameters, and clinical features in patients with MM.

## 5. Conclusion

The current pilot study found a positive association between plasma MCP-1 levels, angiogenesis (expressed as TVA), and the main clinical features of MM (i.e., bone disease, renal dysfunction, and anemia) in newly diagnosed MM patients. However, additional prospective studies with a respectable number of patients should be performed to authenticate the angiogenic potential of MCP-1 and its biological value as a biomarker for monitoring bone or renal disease in patients with MM. The plasma levels of TNF-*α* and IL-6 should also be evaluated parallel to MCP-1.

## Figures and Tables

**Table 1:** Clinical features of patients with multiple myeloma (MM) and healthy volunteers.

Clinical features	Patients with MM (*N* = 45)	Healthy controls (*N* = 24)
Age and sex distribution	Cases	Cases
Male	22	12
Female	23	12
Age (years)	Median 69	Median 67
	Range 44–86	Range 35–83
Plasma cell percentage		
Median	69	
Range	15–97	
Durie-Salmon stage	Cases	
I	8	
II	9	
III	28	
Renal dysfunction	Cases	
Yes	16	
No	28	
Anemia	Cases	
Yes	35	
No	9	
Bone disease	Cases	
Yes	31	
No	14	

Renal dysfunction = serum creatinine level above the upper limit of normal; anemia = hemoglobin value 20 g/L below the lower limit of normal; bone disease = presence of any lytic lesion or severe osteopenia with compressive fractures on standard bone radiographs.

**Table 2:** Measured plasma MCP-1 levels based on clinical parameters in patients with MM.

Clinical feature	MCP-1 (pg/mL)		
	Median		Range
Renal dysfunction			
No	91.5		8.3–277.4
Yes	120.3		84.7–299.5
*p* value		**0.02 **	
Anemia			
No	78.9		8.3–170.2
Yes	109.5		32.1–299.5
*p* value		**0.04 **	
Bone disease			
No	86.4		8.3–138.9
Yes	110.3		32.1–299.5
*p* value		**0.01 **	

*p* values are based on the Mann-Whitney *U* test.

Renal dysfunction = serum creatinine level above the upper limit of normal; anemia = hemoglobin value 20 g/L below the lower limit of normal; bone disease = presence of any lytic lesion or severe osteopenia with compressive fractures on standard bone radiographs.

**Table 3:** Correlation between plasma MCP-1 levels and analyzed parameters of angiogenesis in MM patients.

		Parameter of angiogenesis	
		MVD	TVA
MCP-1 (pg/mL)	*r* ^1^	0.207	0.347
	*p* ^1^	0.18	0.02

^1^Pearson correlation.

MVD = total count of microvessels per 1 mm^2^; TVA = total area occupied by microvessels (as percentage of total section area).
